# Antibacterial activity of jalapeño pepper (*Capsicum annuum* var. *annuum*) extract fractions against select foodborne pathogens

**DOI:** 10.1002/fsn3.453

**Published:** 2017-02-13

**Authors:** Karleigh Bacon, Renee Boyer, Cynthia Denbow, Sean O'Keefe, Andrew Neilson, Robert Williams

**Affiliations:** ^1^ Department of Food Science and TechnologyHuman Agriculture Biosciences BuildingBlacksburgVAUSA; ^2^ Department of Plant Pathology, Physiology, and Weed ScienceVirginia Polytechnic Institute and State UniversityBlacksburgVAUSA; ^3^ Department of Food Science and Technology1013 Integrated Life Sciences Building 1BlacksburgVAUSA

**Keywords:** antimicrobial activity, *Capsicum annuum*, foodborne pathogens

## Abstract

*Capsicum annuum* fruits have been investigated for antimicrobial activity in a number of studies. Capsaicin or other cinnamic acid pathway intermediates are often suggested to be the antimicrobial component, however there are conflicting results. No research has specifically fractionated jalapeño pepper (*Capsicum annuum* var. *annuum*) extract to isolate and identify compound(s) responsible for inhibition. In this study, fractions were collected from jalapeño pepper extracts using reverse‐phase HPLC and tested for antibacterial activity using the disk diffusion method. Following initial fractionation, two fractions (E and F) displayed antibacterial activity against all three pathogens (*p *>* *.05). Commercial standards were screened to determine when they elude and it was found that capsaicin elutes at the same time as fraction E. Fractions E and F were subject to further HPLC fractionation and antibacterial analysis using two methods. The only fraction to display clear inhibition using both was fraction E1, inhibiting the growth of *L. monocytogenes*. Fraction E1 was analyzed using HPLC‐MS. The resulting mass spectra revealed fraction E1 contained compounds belonging to a group of *C. annuum*‐specific compounds known as capsianosides. Limited research is available on antibacterial activity of capsianosides, and a pure commercial standard is not available. In order to confirm the potential antimicrobial activity of the compound(s) isolated, methods need to be developed to isolate and purify capsianosides specifically from jalapeño peppers.

## 1 Introduction

Since the discovery of antibiotics in the 1950s, the use of plant‐derived antiseptics has been comparatively nonexistent. With the development of many antibiotic‐resistant strains of microorganisms (Andersson, 2003; Longenecker & Oppenheimer, 1982; Montravers et al., 1996) and a trending consumer distrust of “unnatural” food ingredients, a resurgence of interest in natural antimicrobials in the food industry has occurred. At present, foods are typically preserved by compounds such as nitrite, sodium benzoate, and sodium metabisulfite that have been tested and proven safe (Gould & Russell, 2003). However, there are occasional reports of allergic reactions to these preservatives, and even potential formation of carcinogenic by‐products like nitrosamines from nitrite (Roller, 2003).

Plants and herbs contain many different classes of phytochemicals (Dorman & Deans, 2000). These phytochemicals include terpenoids, alkaloids, lectins, polypeptides, quinones, phenolics, flavonoids, coumarins, and others (Cowan, 1999). There is an abundance of research in microbiology focused on plant essential oils and their ability to inhibit spoilage and pathogenic food bacteria (Altundag, Aslim, & Ozturk, 2011; Bevilacqua, Gallo, Perricone, Corbo, & Singaglia, 2010; Du, Olsen, Avena‐Bustillos, Friedman, & McHugh, 2011; El‐Baroty, Farag, Abd‐El‐Baky, & Saleh, 2010; Gao et al., 2011; Hsouna et al., 2011; Lazarevic, Dordevic, Zlatkovic, Radulovic, & Palic, 2011; Lee, Jang, Deo, & Kim, 2011; Prakash et al., 2011; Rahman, Bajpai, Nguyen, & Sun, 2011; Sanchez‐Gonzalez et al., 2011; Serrano et al., 2011; Viazis, Akhtar, Feirtag, & Diez‐Gonzalez, 2011). Essential oils isolated from plant sources have been found to be effective antimicrobial agents, and there is ongoing research to identify more antimicrobial plant sources.

Extracts from *Capsicum annuum* fruit have been investigated to some extent, and antimicrobial properties have been reported with mixed results. Crude tissue extracts from several different *C. annuum* varieties have inhibited growth of species of *Bacillus*,* Clostridium*,* Pseudomonas*,* Listeria*,* Salmonella*,* Staphylococcus*, and *Streptococcus* (Bacon et al., 2016; Careaga et al., 2003; Cichewicz & Thorpe, 1996; Dorantes et al., 2000). Extract from jalapeno fruit, specifically, has inhibited *Streptococcus pyogenes*,* Listeria monocytogenes*,* Clostridium sporogenes*, and *Clostridium tetani* (Bacon et al., 2016; Cichewicz & Thorpe, 1996). However, when trials were completed comparing results to a commercial capsaicin product (60% and 98% purity), there was no antimicrobial activity reported suggesting activity is not exclusively associated with the capsaicin in the pepper (Bacon et al., 2016; Cichewicz & Thorpe, 1996). Another study did, however, report that high concentrations of a commercial capsaicin were inhibitory against *Bacillus subtilis*, specifically (Molina‐Torres, Garcia‐Chávez, & Ramirez‐Chávez, 1999).

Dorantes et al. (2000) separated the extracts from three different chili peppers (habanero, serrano, and pimento) using reverse‐phase high‐performance liquid chromatography (HPLC) and the antimicrobial activity of the extracted capaisinoid compounds was determined. Capsaicin and dihydrocapsaicin were not inhibitory, but coumaric and cinnamic acids were (Dorantes et al., 2000). Our research group previously fractionated *C. annum* var. jalapeno extract to determine the best method for extraction and found that the fractions eluded between 20 and 30 min contained the most antimicrobial activity, especially against *L. monocytogenes* (Bacon et al., 2016). To our knowledge, there have been no other studies that have evaluated the fractions of *C. annum* var. jalapeno for antimicrobial activity. The purpose of this study was to further investigate the fractions of *C. annum* var. jalapeno previously determined to possess the greatest antimicrobial activity.

## 2 Materials and Methods

### 2.1 Preparation of jalapeño pepper extract

Extract from jalapeño peppers (*C. annuum* var. *annuum*, Jalapeño, cv. “Del Mar”) was prepared as described by Bacon et al. (2016) using no solvent. Any extract that was not used immediately was stored at 4°C until use.

### 2.2 Fractionation of jalapeño extracts using high‐performance liquid chromatography

Crude jalapeno extract was initially fractionated as described by Bacon et al. (2016). The HPLC schedule is shown in Table 1A. After the initial screening against the three pathogens, fractions E and F were selected for further fractionation using a different HPLC setup. For this portion of the study, HPLC equipment used was the same, but the acetonitrile gradient was changed consisting of two solvents: solvent A (0.1% aqueous acetic acid in water) and solvent B (0.1% acetic acid in acetonitrile) was utilized. The HPLC schedule for fractions E and F can be seen in Table 1B and C, respectively. Fractions were collected automatically based on peak slope (which is a different method than we used to collect fractions of the crude extract) and sorted into fraction collection vials (Agilent, Santa Clara, CA). Nomenclature for fractions collected were assigned based on retention time and peak elution. Fractions were pooled into sterile glass tubes and processed as described by Bacon et al. (2016) for antimicrobial assays. Additionally, commercial standards of phenylalanine, cinnamic acid, coumaric acid, caffeic acid, vanillin, and capsaicin (Sigma‐Aldrich, St. Louis, MO) dissolved in 95% aqueous ethanol were used to determine separation characteristics of jalapeño extract. Peaks from known standards were identified by retention time and UV absorbance spectra.

**Table 1:** HPLC gradient schedule for analysis of jalapeño extracts

Time (min)	%A^a^	%B^b^
(A) HPLC gradient schedule for whole jalapeño extract		
0	100	0
35	38	62
40	0	100
41	100	0
45	100	0
(B) HPLC gradient schedule for fraction E		
0	60	40
10	48	52
11	0	100
12	0	100
15	60	40
(C) HPLC gradient schedule for fraction F		
0	50	50
10	40	60
11	0	100
12	0	100
15	50	
(D) HPLC gradient schedule for LC‐MS analysis of fraction E1		
0	99.0	1.0
10	99.0	1.0
30	5.0	95.0
40	5.0	95.0
50	99.0	1.0

a Solvent A = 0.1% aqueous acetic acid in water.

b Solvent B = 0.1% acetic acid in acetonitrile.

### 2.3 Bacterial cultures and conditions

Three bacterial cultures were used in this study: *L. monocytogenes* V7 (provided by CDC, Atlanta, GA), *Escherichia coli* O157:H7 (obtained from a cider outbreak and provided by Dr. L. R. Beuchat from the University of Georgia, Griffin, GA), and *Salmonella enterica* Baildon (obtained from a lettuce/tomato outbreak and provided by Dr. L. R. Beuchat from the University of Georgia, Griffin, GA). Cultures were prepared and culture identification methods utilized have been described previously by Bacon et al. (2016).

### 2.4 Antimicrobial assays

The disk diffusion assay and statistical analysis were performed as described in Bacon et al. (2016) with no modifications. Initial fractions (A–G) were evaluated against growth of all three pathogens. However, only growth of *L. monocytogenes* was screened with the subsequent fractions of E and F. Two plates were prepared for each pathogen/extract fraction combination (*n* = 4). Each experiment was replicated three times (*N* = 12).

Subfractions were also evaluated using the automated growth curve analysis method to further confirm inhibition (where identified). This was completed using a Bioscreen C Microbiology Reader (Growth Curves, Piscataway, NJ) equipped with an incubator and automated turbidimeter to determine the optical density (OD) of the culture over time. The Bioscreen measures microbial growth by vertical pathway, and the changes in optical density in liquid medium are correlated with microbial populations in the samples. OD was determined between 420 and 540 nm. The liquid growth medium used was either 0.1% buffered peptone water or TSB. Each well of a honeycomb microwell plate (Bioscreen, Growth Curves, Piscataway, NJ) was filled with 125 μl of growth medium (BPW or TSB), 15 μl of prepared jalapeño extract fraction, and 10 μl of culture (containing approximately 10^3^ cells). For controls, eluent was collected from the HPLC column using the same conditions as the experimental fraction, but water was injected into the column rather than jalapeño extract, and processed as described above. Control wells contained 15 μl of corresponding fraction controls to replace jalapeño extract. Microwell plates were incubated at 37°C for either 24 hr (peptone) or 72 hr (TSB), and OD was measured every 15 min with 10 s of shaking before each reading. The growth curve data were generated by using EZ Experiment software (Growth Curves, Piscataway, NJ) and exported as a Microsoft Excel spreadsheet (Microsoft, Seattle, WA). Each experiment was replicated three times. Growth curves generated were analyzed for statistical difference of the means of trapezoidal area under the curve (AUC) values using Student's *t*‐test. All statistical analyses were performed with JMP 7.0 software (SAS Institute, Cary, NC) and the difference was set to be significant when the *p*‐value was less than 0.05.

### 2.5 HPLC–mass spectrometry analysis

Fraction E1, which showed antilisterial activity in our antimicrobial experiments, was chosen for further analysis using HPLC‐MS. An Agilent (Palo Alto, CA) 1100 series HPLC coupled to a triple quadrupole mass spectrometer API 3200 (Applied Biosystems Sciex Instruments, Rotterdam, the Netherlands) equipped with a Turbo Ion Spray interface (Electrospray) was used for the analysis. HPLC separations were accomplished using a Kinetex (Phenomenex Torrance, CA), C18 (3 × 100 mm, 2.6 μm) column. A gradient was used consisting of solvent A (deionized water with 0.1% acetic acid) and solvent B (acetonitrile with 0.1% acetic acid), and a flow rate of 0.4 ml/min was used throughout the analysis. The gradient schedule is listed in Table 1D. The injection volume was 10 μl. The gradient was run for 30 min. Both positive and negative ion mass spectra were recorded. The MS operating parameters were as follows: capillary voltage (IS) ±4.2 kV, declustering potential (DP) −50 V, and source temperature set to 420°C. A full scan of mass spectra from m/z 300 to 1,800 was performed. After initial MS analysis, there was consistent appearance of high mass spec ions near 10 min into the gradient, and the method was altered to look only at m/z 900 to 1,400.

## 3 Results and Discussion

**Figure 1:**
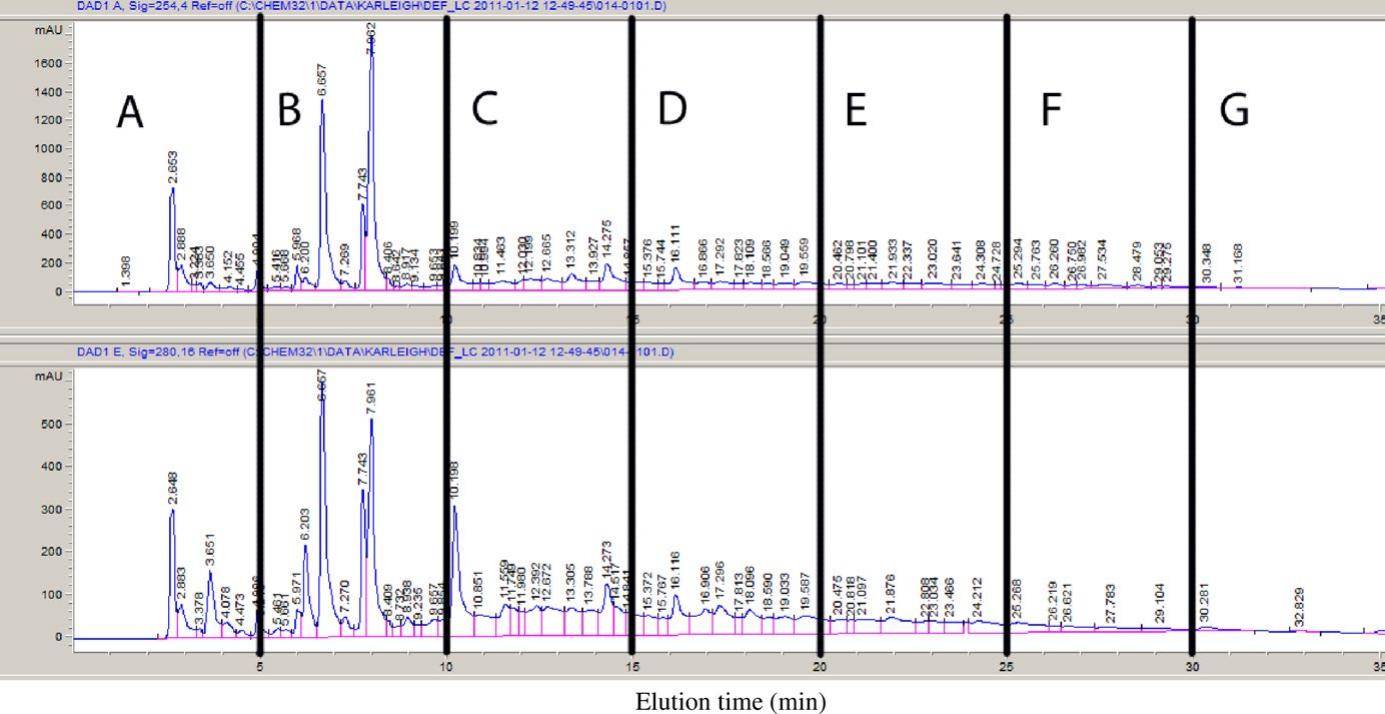
Reverse‐phase HPLC UV chromatograms (top: 254 nm, bottom: 280 nm) of jalapeño extract with vertical indicators of fractions collected. Fractions were collected every 5 min and assigned alphabetical labels based on time of elution (Bacon et al., 2016)

**Figure 2:**
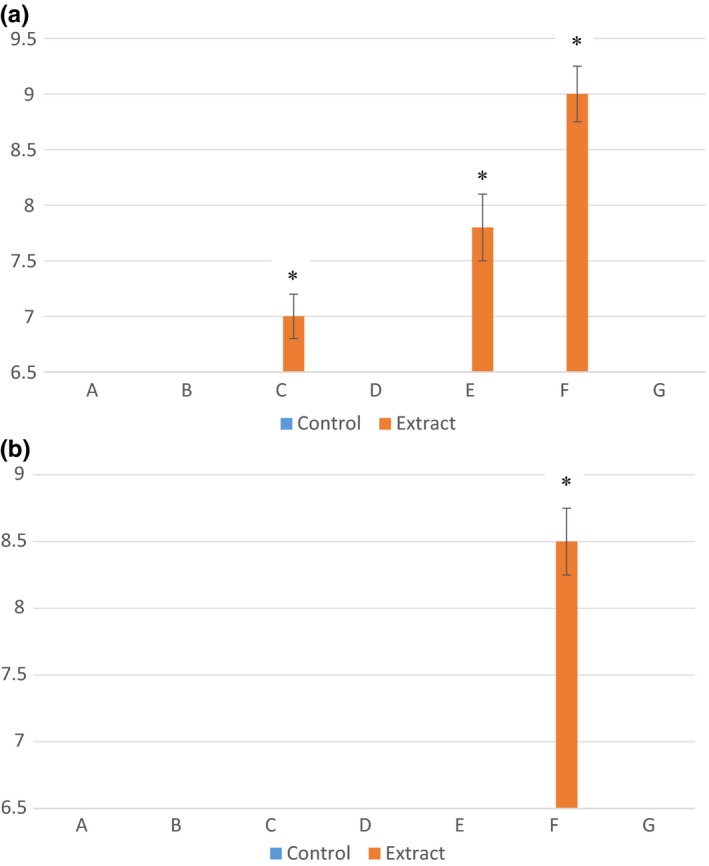
Mean zones of inhibition measurements (mm) for bacterial cultures (a) *Listeria monocytogenes* and (b) *Salmonella enterica* in association with fractions (A–G) of crude jalapeño extract. Error bars represent standard error of the mean. An asterisk indicates significant difference between the mean zones of inhibition observed and control for the same fraction. If no inhibition was seen, a value of 6.5 was assigned, which was the diameter of the disk used for the disk diffusion experiments

A chromatogram of the initial fractionation of jalapeno extract is provided in Figure 1. *Listeria monocytogenes* was most susceptible to the fractionated extracts, with zones of inhibition larger (*p *<* *.05) than the control for fractions C, E, and F (Figure 2). *Escherichia coli* O157:H7 was not inhibited at all by the extracts, and only one fraction (F) significantly inhibited growth of *Salmonella* (*p *<* *.05), therefore no further evaluation of the extracts against these pathogens was completed (Figure 2).

### 3.1 HPLC analysis of known phenylpropanoid standard compounds

A number of known phenylpropanoid standards (phenylalanine, caffeic acid, coumaric acid, vanillin, cinnamic acid, and capsaicin) were analyzed using the same reverse‐phase HPLC conditions, and the elution times were determined (Table 2). Only cinnamic acid and caffeic acid eluted at time points that would place them in a collected fraction was considered “active.” Since fraction C only displayed minimal inhibition, it was not evaluated further. Cinnamic acid, eluted at 23.60 ± 0.16 min, placing the compound in fraction E, displayed the greatest inhibition. It is well‐known that cinnamic acid possesses antimicrobial activity against both bacteria and fungi (Nascimento, Locatelli, Freitas, & Silva, 2000; Olasupo, Fitzgerald, Gasson, & Narbad, 2003; Roller & Seedhar, 2002; Wen, Delaquis, Stanich, & Toivonen, 2003; Yao & Shelef, 1998). The minimum inhibitory concentration (MIC) of cinnamic acid for *L. monocytogenes* is between 1,000 and 2,000 ppm (0.1%–0.2%) in acidic conditions (Wen et al., 2003; Yao & Shelef, 1998). The pH of the crude jalapeno extract was determined to be 5.67 (Bacon et al., 2016). Our extract contained approximately 5 ppm of cinnamic acid according to quantification using an external standard curve; therefore, it is unexpected that the presence of this compound in the subfraction contributed was a key component in the inhibition (data not shown).

**Table 2:** Reverse‐phase HPLC elution times of known phenylpropanoid intermediates produced during the cinnamic acid pathway for collecting fractions A through G (*n* = 3)

Compound	Elution time (min)	Fraction theoretically contained in
Phenylalanine	5.964 ± 0.003	B
Caffeic acid	13.471 ± 0.170	C
Coumaric acid	15.941 ± 0.208	D
Vanillin	16.187 ± 0.065	D
Cinnamic acid	23.595 ± 0.155	E
Capsaicin	32.801 ± 0.053	G

### 3.2 Effect of subfractions E and F on the growth of foodborne pathogens

**Figure 3:**
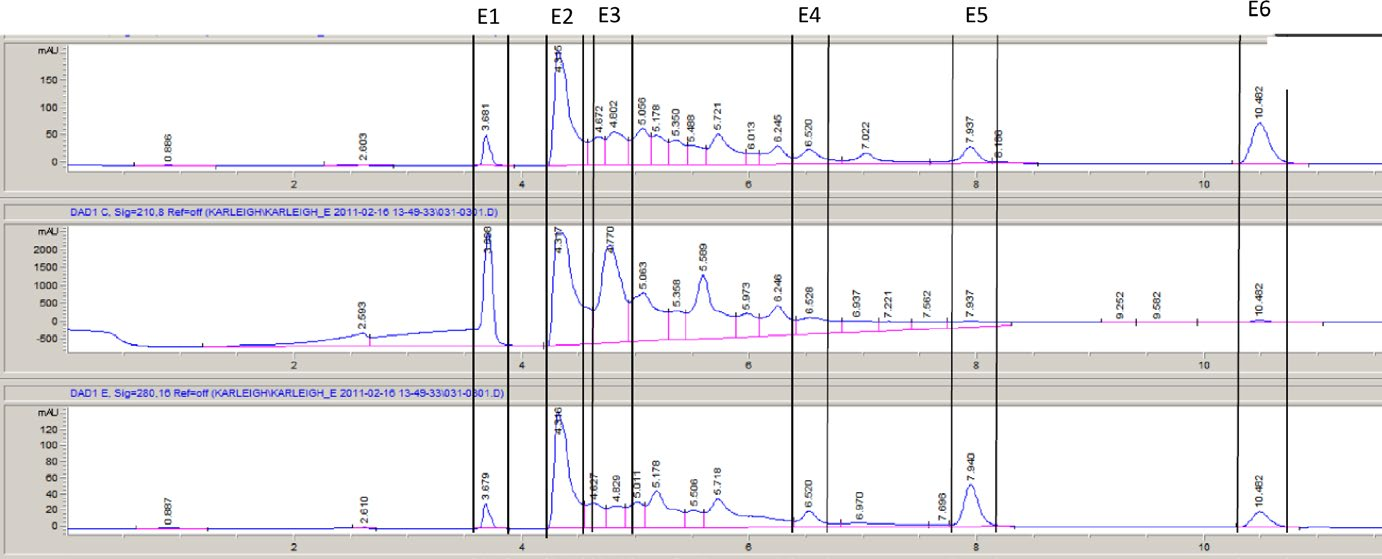
Reverse‐phase HPLC chromatogram of jalapeño extract fraction E with vertical indicators of fraction collected. Fractions were collected based on a peak‐by‐peak basis and assigned numerical labels based on time of elution

**Figure 4:**
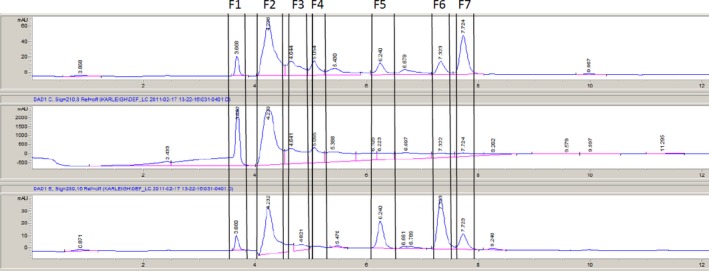
Reverse‐phase HPLC chromatogram of jalapeño extract fraction F with vertical indicators of fraction collected. Fractions were collected based on a peak‐by‐peak basis and assigned numerical labels based on time of elution

Since fractions E and F were the most active, they were further fractionated to investigate active compounds further. These fractions were collected on a peak‐by‐peak basis rather than by time as was used to collect fractions A through G. It is likely that there is more than one compound in each peak collected, but the compounds within each peak are expected to be similar to each other in terms of polarity. Six subfractions were collected from fraction E, labeled in numerical order as they eluted (1–6). Seven subfractions were collected from fraction F, labeled in numerical order as they eluted (1–7). The chromatograms for these are shown in Figures 3e and 4f.

**Figure 5:**
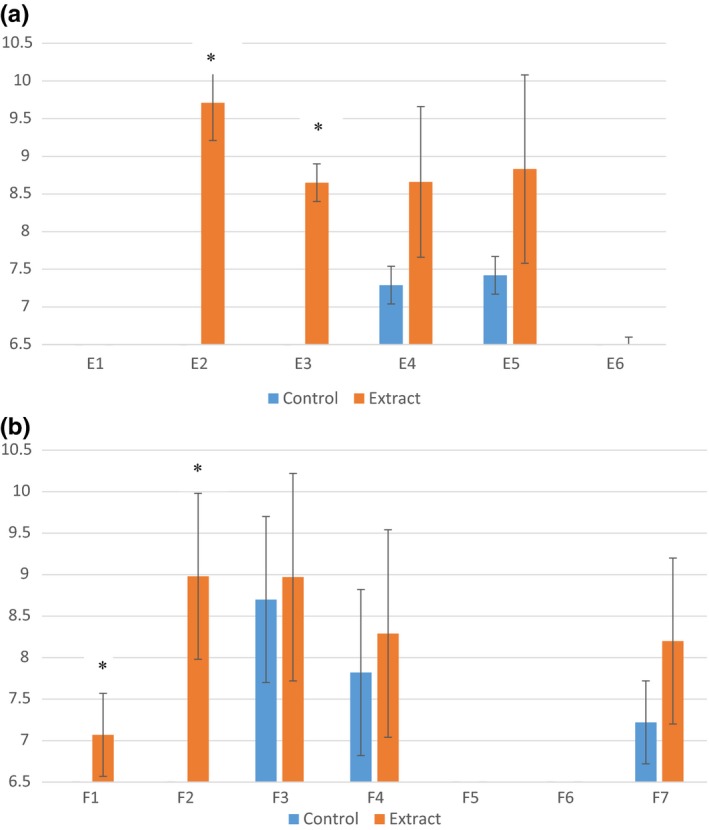
Mean zones of inhibition measurements (mm) for *Listeria monocytogenes* in association with jalapeno extract fraction (a) E subfractions (E1–E6) and (b) F subfractions (F1–F7). Error bars represent standard error of the mean. Asterisk indicates significant difference between the mean zones of inhibition observed and control for the same fraction. If no inhibition was observed, a value of 6.5 was assigned, which is the diameter of the disk used for the experiment

Disk diffusion assays were performed using E and F subfractions against *L. monocytogenes*,* E. coli* O157:H7, and *Salmonella anatum*. The only bacterium with visible zones of inhibition was *L. monocytogenes* (Figure 5). All other bacteria/extract combinations yielded no difference between controls and extracts; therefore, the data are not presented. The E subfractions with significant inhibition were E2 and E3 (*p *<* *.05). E2 had the largest zone of inhibition (Figure 5a). Much like the disk diffusion results for the E subfractions, only *L. monocytogenes* was sensitive to the F subfractions. Exposure to fractions F1 and F2 resulted in zones of inhibition larger than the control (*p *<* *.05) (Figure 5b).

**Figure 6:**
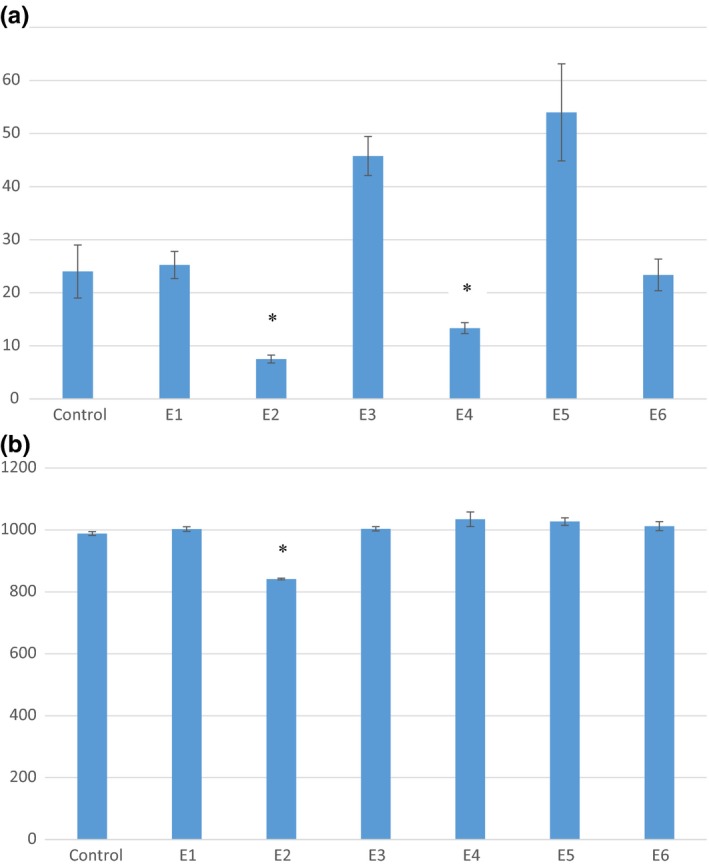
Area under the growth curve comparison for *Listeria monocytogenes* grown in (a) 0.1% peptone and (b) TSB in association with jalapeño extract fractions E1–E6. Error bars represent standard error of the mean. An asterisk indicates mean AUC values that are significantly less that the AUC value of the *L. monocytogenes* control

To further confirm the growth inhibition that was seen using the disk diffusion assay, growth curves were generated and the AUCs for each bacterium/extract combination was calculated. This further confirmed that subfraction E2 was the most inhibitory to growth of the organism in both growth mediums (*p* < .5) (Figure 6). With the exception of subfraction E4 in BPW, the remaining E subfractions did not significantly affect growth of *L*. *monocytogenes* (Figure 5). AUC for E2 was 69% smaller than the control in peptone and 15% smaller than the control in TSB (Figure 6b). As previously reported, the antimicrobial agent cinnamic acid elude in fraction E. The standard was run with HPLC a second time using the same conditions used to subfractionate fraction E. Cinnamic acid was eluted at 7.94 ± 0.02 min. This elution time placed it in fraction E3, which did not display antimicrobial activity for the bacteria tested. The concentration of cinnamic acid in our extract was approximated to be 4 ppm according to a standard curve (data not shown). Although cinnamic acid has been found to have an antimicrobial capacity, the concentration needed was much higher than that of our extract. Therefore, it is not surprising that no antibacterial activity was observed.

The F subfractions had no significant effect on the growth between any of the bacterial cultures (including *L. monocytogenes*) in either growth media (data not shown). Since there were no consistent results using both methods, further analysis was not completed with fraction F. It is concluded that the F fractions lost antimicrobial capacity when subfractionated, and there are a number of possible reasons why this occurred. Antimicrobial compounds can often interact with one another and give either synergistic, additive, or antagonistic effects (Vigil, Palou, Parish, & Davidson, 2005). When additivity occurs, the combined compounds yield antimicrobial activity that is equal to the sum of the activity of the two independent compounds. This is also known as indifference. When compounds are synergistic, they increase or enhance the overall antimicrobial activity beyond that of the sum of the individual compounds. Antagonistic compounds work together to reduce the efficacy of the compounds when compared to their individual results (Vigil et al., 2005). When we tested fraction F, we were testing it with all the compounds that it contained interacting with one another and the result was an inhibitory combination. When the compounds were separated further using HPLC, we removed the compounds' abilities to interact with one another, which resulted in lower inhibition of bacterial growth. Some of the compounds in fraction F were likely working in combination with one another to produce the inhibitory effect. When those compounds were separated, they were not able to produce the same effects they had in combination. For future studies, it may be beneficial to look at combinations of fraction F subfractions to see if the combinations result in increased inhibition.

### 3.3 Mass spectrometry of fraction E2

**Figure 7:**
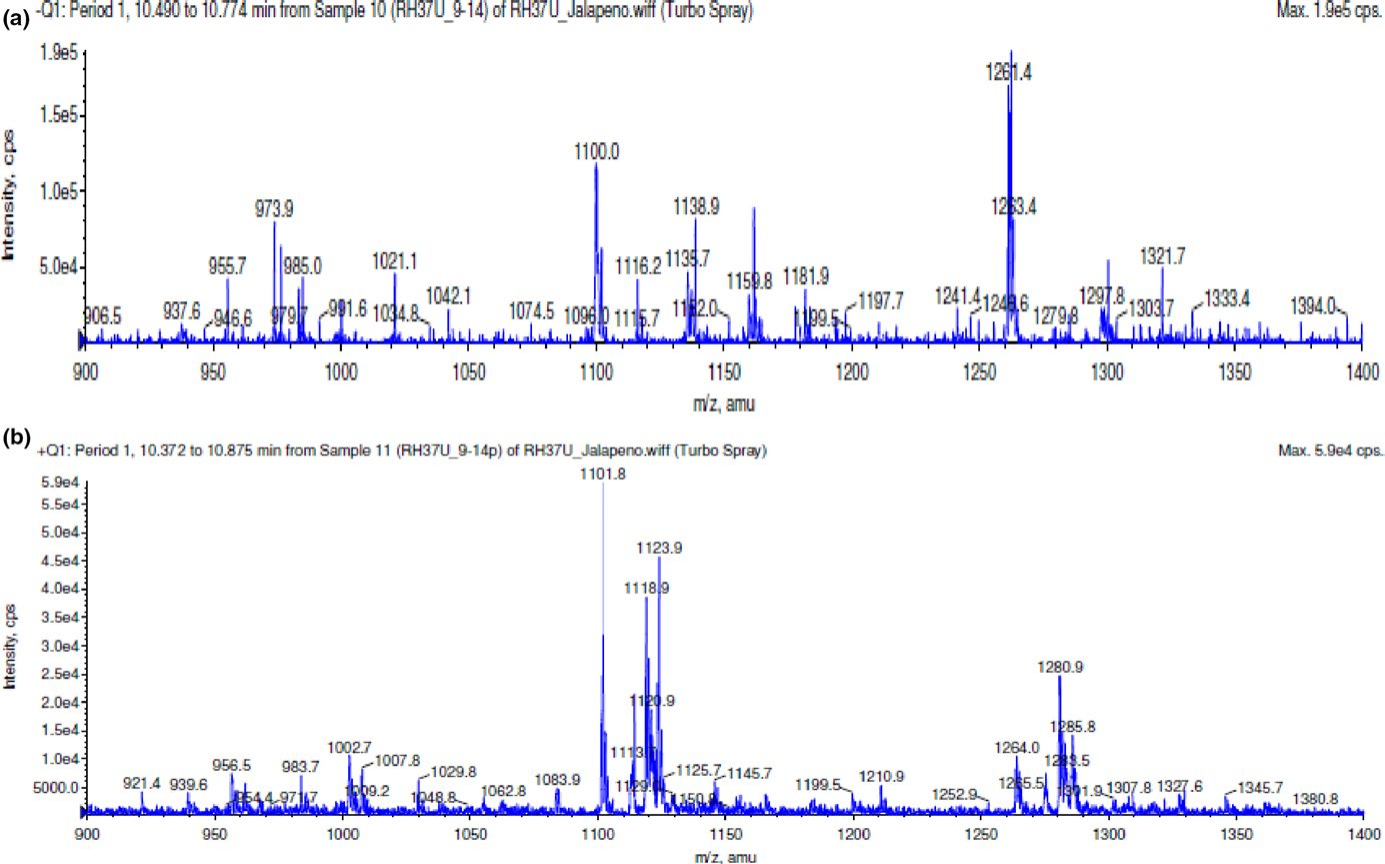
Mass scan of ions contained in fraction E1 from 900 to 1,400 amu. (a) Negative ion scan. (b) Positive ion (+Na) scan

The antibacterial activity of fraction E2 in the presence of *L. monocytogenes* was clearest in this study, therefore mass spectrometry (MS) was performed to identify the compounds present in fraction E1. The initial mass spectrometry runs were performed in both positive and negative ionization modes, scanning a wide mass range (300–1,800 amu). This revealed limited signal. There was a consistent appearance of high mass ions at around 10 min in the gradient, so a method looking at only 900‐1,400 amu (both positive and negative ionization modes) was run. The LCMS data provided are of these analyses (Figure 7). Based on the identified parent ion m/z values (Table 3) and a review of the literature, the predominant compounds found within fraction E1 are likely acyclic diterpene glycosides (De Marino, Iorizzi, & Zollo, 2008; De Marino et al., 2006; Iorizzi, Lanzotti, De Marino, & Zollo, 2001; Iorizzi, Lanzotti, Ranalli, De Marino, & Zollo, 2002; Lee, Kiyota, Ikeda, & Nohara, 2006; Lee, Kiyota, Ikeda, & Nohara, 2007; Lee, Kiyota, Ikeda, & Nohara, 2008; Lee et al., 2009; Materska & Perucka, 2005). There are a number of acyclic diterpene glycosides known as capsianosides that have been isolated specifically from *C. annuum* plants, both sweet and spicy, that share the parent m/z found in our MS analysis (Table 3). Several known capsianosides have parent ion m/z values that match with the values we discovered in our MS scan. In order to make a positive identification of the compounds present in fraction E1, either a known standard would need to be purchased and MS analysis run in parallel or a pure compound needs to be isolated and analyzed by MS and NMR. Capsianoside standards are not currently commercially available. Therefore, we were not able to verify our mass spec results with positive capsianoside controls or obtain an absolute identification. A limited number of studies have been conducted on biological activities of capsianosides. The antimicrobial activity of capsianoside II has been screened using an agar dilution assay against both yeast and fungi with negative results (Iorizzi et al., 2002). Furthermore, antioxidant activity of capsianosides VIII and III was tested with negative results (De Marino et al., 2006). Increased permeability in cellular tight junctions has been reported with exposure of cells to some capsianosides without causing toxicity to the cells (Hashimoto, Kawagishi, Nakayama, & Shimizu, 1997; Shimizu, 2010). It has also been shown that capsianoside F has Ca^2+^ chelating activities that are about one‐tenth that of EDTA (Hashimoto et al., 1997).

**Table 3:** Ion m/z detected in fraction E1 MS scans that correlate to capsianosides isolated and identified in the literature by MS analysis

Parent ion (m/z)	Tentative ID	Reported parent ion (m/z) a
937.9 [M − H]^−^	Capsianoside IX	937 [M − H]^−^ b
	Capsianoside XVII	961.47 [M + Na]^+^ e
1,123.9 [M + Na]^+^ 1,100.0 [M − H]^−^ 1,101.8 [M + Na]^+^	Capsianoside III	1,123.51 [M + Na]^+^ b ^,^ d
	Capsianoside VIII	1,123.53 [M + Na]^+^ c
	Capsianoside IX	1,123.53 [M + Na]^+^ c
	Capsianoside XV	1,123 [M + Na]^−^ c
1,285.8 [M + Na]^+^	Capsianoside X	1,285.59 [M + Na]^+^ c
	Capsianoside XVIII	1,285.57 [M + Na]^+^ f

a Letter following corresponds to the publication in which the MS data are published.

b De Marino et al. (2006).

c Lee et al. (2006).

d Lee et al. (2007).

e Lee et al. (2008).

f Lee et al. (2009).

Calcium is important for growth of several bacteria (Arakawa, Saito, Saito, Kakegawa, & Kobayashi, 2000; Norris, Onoda, Pollaert, & Grehan, 1999; Norris et al., 1991; Onoda et al., 2000; Perry & Brubaker, 1987; Smith, 1995). Compounds that chelate calcium have successfully been used to disrupt biofilm formation and prevent biofilm‐related infections (Banin, Brady, & Greenberg, 2006; Ozerdem, Elci, Atmaca, Akbayin, & Gul, 2003; Percival et al., 2005; Raad et al., 2008). The presence of Ca^2+^ and other cations in the peptidoglycan layer of Gram‐positive bacteria like *L. monocytogenes* is necessary to provide the correct ionic environment for cation‐dependent membrane transport systems (Hughs, Hancock, & Baddiley, 1973). Gram‐negative microorganisms, such as *E. coli* O157:H7 and *S. enterica*, do not have the cell wall requirement for calcium (Ferris, 1989). This difference in calcium requirement between Gram‐positive and Gram‐negative bacteria may explain why there was inhibition of *L. monocytogenes* in the presence of fraction E1, while *E. coli* O157:H7 and *S. enterica* were not affected. For this statement to be true, however, two major assumptions must be made: (1) fraction E1 does in fact contain capsianoside(s) and (2) the capsianoside(s) present possess calcium chelating abilities.

## 4 Conclusions

Jalapeño extract has been examined in the past for antibacterial activity. Although some have reported that capsaicin and other cinnamic acid pathway intermediates are responsible for antimicrobial activity, no research had been conducted that include fractionating the extracts and isolating the compounds responsible for inhibition. This study showed that at least one of the resulting subfractions from crude jalapeño extract consistently inhibited growth of *L. monocytogenes* using two different experimental methods. Upon HPLC‐MS analysis, the m/z values of ions present in the subfractions (E1) matched those of a known group of *C. annuum*‐specific compounds known as capsianosides. Little information is known about capsianosides. No commercial capsianoside standard is available for MS analysis; therefore, no positive control could be run to confirm the identity. A method should be developed to isolate capsianosides specifically from jalapeño peppers and purify the extract so that further analysis can be performed.

## Conflict of Interest

None declared.
